# Assessment of Non-Invasive Measurements of Oxygen Saturation and Heart Rate with an Apple Smartwatch: Comparison with a Standard Pulse Oximeter

**DOI:** 10.3390/jcm11061467

**Published:** 2022-03-08

**Authors:** Carmen Spaccarotella, Alberto Polimeni, Cinzia Mancuso, Girolamo Pelaia, Giovanni Esposito, Ciro Indolfi

**Affiliations:** 1 Division of Cardiology, Department of Advanced Biomedical Science, Federico II University, 80138 Naples, Italy; carmenspaccarotella@gmail.com (C.S.); espogiov@unina.it (G.E.); 2 Center for Cardiovascular Research, Division of Cardiology, Department of Surgical and Medical Sciences, University Magna Graecia, 88100 Catanzaro, Italy; polimeni@unicz.it (A.P.); cinzia_mancuso@live.it (C.M.); 3 Department of Health Sciences, University Magna Graecia, 88100 Catanzaro, Italy; pelaia@unicz.it; 4 Mediterranea Cardiocentro, 80138 Naples, Italy

**Keywords:** Apple Watch 6, COVID-19, SpO_2_

## Abstract

The most commonly used method to assess peripheral oxygen saturation (SpO_2_) in clinical practice is pulse oximetry. The smartwatch Apple Watch 6 was developed with a new sensor and an app that allows taking on-demand readings of blood oxygen and background readings, day and night. The present study aimed to assess the feasibility and agreement of the Apple Watch 6 compared with a standard SpO_2_ monitoring system to assess normal and pathological oxygen saturation. We recruited study participants with lung disease or cardiovascular disease and healthy subjects. A total of 265 subjects were screened for enrolment in this study. We observed a strong positive correlation between the smartwatch and the standard commercial device in the evaluation of SpO_2_ measurements (r = 0.89, *p* < 0.0001) and HR measurements (r = 0.98, *p* < 0.0001). A very good concordance was found between SpO_2_ (bias, −0.2289; SD, 1.66; lower limit, −3.49; and upper limit, 3.04) and HR (bias, −0.1052; SD, 2.93; lower limit, −5.84; and upper limit, 5.63) measured by the smartwatch in comparison with the standard commercial device using Bland–Altman analysis. We observed similar agreements and concordance even in the different subgroups. In conclusion, our study demonstrates that the wearable device used in the present study could be used to assess SpO_2_ in patients with cardiovascular or lung diseases and in healthy subjects.

## 1. Introduction

The most widely used method to assess peripheral oxygen saturation (SpO_2_) in clinical practice is pulse oximetry. The major advantage of this method is that it is non-invasive and has other benefits such as ease of use so that it can be used for multiple out-of-hospital measurements [1]. For these reasons, pulse oximetry is also often used in patients with COVID-19 to monitor peripheral oxygen saturation frequently [2]. Standard pulse oximetry measures the oxygen saturation in the blood by shining light at specific wavelengths through tissue. Deoxygenated and oxygenated hemoglobin absorb light at different wavelengths (660 nm and 940 nm, respectively), and an algorithm processes the absorbed light in the pulse oximeter to display a saturation value. The smartwatch Apple Watch 6 was developed with a new sensor and an app that allow taking on-demand readings of blood oxygen and background readings, day and night [3]. Smartwatches are widespread and are increasingly being used for digital health information. For instance, the Apple Watch can reliably detect atrial fibrillation [4,5,6], and we previously showed the possibility of using this smartwatch to obtain multiple ECG leads [7] to detect ST-segment ECG changes. More recently, we and others demonstrated that it is possible to assess QTc measurements with the Apple Watch [8,9] and even Brugada syndrome ECG patterns [10]. Accordingly, the present study aimed to assess the feasibility and agreement of the Apple Watch 6 compared with a standard SpO_2_ monitoring system to assess normal and pathological oxygen saturation in a large cohort of patients with cardiovascular disease, patients with lung disease and healthy subjects.

## 2. Materials and Methods

We recruited study participants older than 18 years with lung disease or cardiovascular disease. A group of healthy subjects was also included for comparison. Exclusion criteria were as follows: (1) missing upper extremity, hand, or finger; (2) inability to wear a watch because of wrist circumference or edema of the arm, wrist, or hand; (3) clinical instability.

The Ethical Committee of the University Magna Graecia approved the study, and all subjects included in this study gave their written informed consent. The study conforms to the principles outlined in the Declaration of Helsinki and is independent from industry.

Research staff were trained to measure SpO_2_ and HR with a smartwatch and a standard pulse oximeter according to their manufacturers’ guidelines. The SpO_2_ and HR measurements were obtained with the Apple Watch 6 (Apple Inc., Cupertino, CA, USA) and with a standard Nellcor Portable SpO_2_ Patient Monitoring System, PM10N (Medtronic, Minneapolis, MN, USA), placed on the index and middle fingers of the left hand (the same arm that was used for smartwatch measurement). The measurement accuracy of the latter system is ±2 digits in the range of values from 70% to 100% for saturation, and ±3 digits in the range of values from 20 to 250 bpm for pulse rate [11]. The measurements with the Apple Watch 6 and the Nellcor system were taken within 1 minute of each other in order to ensure comparability between the two devices. All subjects followed the same measurement schedule, and all measurements were repeated two times and averaged. Since movement artifacts are factors that can affect the reliability of measurements when using the smartwatch, particular attention was paid to its correct placement. During the measurements, the arms were placed at rest on a table. The wrist and palm were placed face down on a flat surface and held steady. Particular attention was paid to ensuring that the Apple Watch fitted snugly against the wrist. The wristband was snug but comfortable, and the back of the Apple Watch touched the wrist. If the wrist bones prevented the watch from fitting snugly, it was moved along the arm to about 2.5 to 5 cm above the wrist [12].

The primary aim of the study was the head-to-head comparison of the measurements of SpO_2_ and HR by the smartwatch and the standard pulse oximeter. The secondary aim was the comparison of the measurements of SpO_2_ and HR between subgroups (lung disease, CV disease, healthy subjects).

Continuous variables are presented as mean ± standard deviation. For the assessment of differences in metric outcome variables, we used paired *t*-tests, and in the case of binary variables we used chi-square tests. A one-way analysis of variance (ANOVA) was used to determine any statistically significant differences between the means of two or more independent (unrelated) groups. A *p*-value of <0.05 was considered statistically significant. Shapiro–Wilk tests were used to assess the normality of continuous variables. The correlation between the two technologies was assessed using linear regression and estimated with Pearson analysis for normally distributed data and Spearman analysis for nonparametric data [13]. A plot of the differences between techniques was created according to the method described by J.M. Bland and D.G. Altmann [14]. Statistical analyses were performed using MedCalc Statistical Software, version 14.8.1 (MedCalc Software, Ostend, Belgium), and GraphPad Prism, version 8.0.0 (GraphPad Software, San Diego, CA, USA).

## 3. Results

A total of 265 subjects were screened for enrolment in this study. Three subjects were excluded from the study as it was impossible to obtain data from them, probably due to them having small wrists. In five subjects, it was not possible to assess oxygen saturation with the Apple Watch despite multiple attempts, for reasons that could not be detected. After screening, 257 subjects were included in the present study. Of these 257 subjects, 56 were healthy controls, 60 were patients with lung disease, and 141 were patients with cardiovascular disease. The study population is described in Table 1.

**Figure 1:**
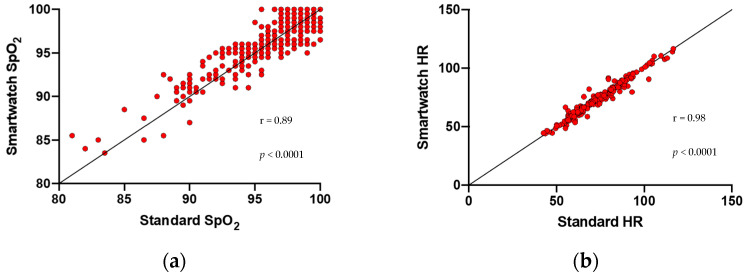
Correlation plots between the smartwatch and the standard commercial device. (**a**) Correlation between the smartwatch and the standard commercial device in the evaluation of SpO_2_ measurements (r = 0.89). (**b**) Correlation between the smartwatch and the standard commercial device in the evaluation of HR measurements (r = 0.98).

Healthy subjects were younger than patients with lung or CV disease (*p* < 0.001) and had fewer risk factors. No differences were found regarding the technical features of measurements (room temperature, body temperature, wrist circumference; *p* = NS). We observed strong positive correlations between the smartwatch and the standard commercial device in the evaluation of SpO_2_ measurements (r = 0.89, *p* < 0.0001) and HR measurements (r = 0.98, *p* < 0.0001) (Figure 1a,b).

**Figure 2:**
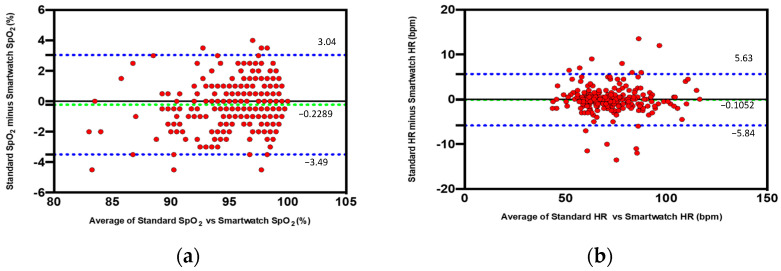
Concordance between SpO_2_ and HR measured by the smartwatch in comparison with the standard commercial device using Bland–Altman analysis. Bland–Altman plots indicate the level of agreement between the smartwatch and the standard commercial device. The dashed green line represents the bias (mean difference), and the dashed blue lines represents the upper and the lower limits of agreement. This difference is considered clinically nonsignificant. (**a**) Difference in % of SpO_2_ between the smartwatch and the standard commercial device plotted against the mean of the two readings. (**b**) Difference in beats per minute in HR between the smartwatch and the standard commercial device plotted against the mean of the two readings.

A very good concordance was found between SpO_2_ measured by the smartwatch in comparison with the standard commercial device (bias, −0.2289; SD, 1.66; lower limit, −3.49; and upper limit, 3.04) using Bland–Altman analysis. Figure 2a shows the difference in % of SpO_2_ between the smartwatch and the standard commercial device plotted against the mean of the two readings. This difference was considered clinically nonsignificant. Similarly, an excellent agreement was found between HR measured by the smartwatch in comparison with the standard commercial device (bias, −0.1052; SD, 2.93; lower limit, −5.84; and upper limit, 5.63) using Bland–Altman analysis. Figure 2b shows the difference in beats per minute in HR between the smartwatch and the standard commercial device plotted against the mean of the two readings. This difference was considered clinically nonsignificant.

**Figure 3:**
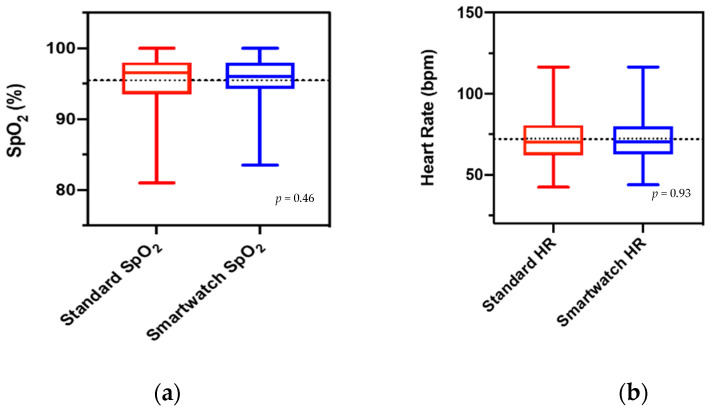
Boxplots of the mean differences between the smartwatch and the standard commercial device. (**a**) Mean difference in SpO_2_ between the smartwatch and the standard commercial device (*p* = 0.46) (**b**) Mean difference in HR between the smartwatch and the standard commercial device. (*p* = 0.93).

Furthermore, based on the mean differences between the smartwatch and the standard commercial device, no statistically significant differences were found in both SpO_2_ and HR measurements (*p* = 0.46 and *p* = 0.93, respectively) (Figure 3a,b).

We observed similar agreements and concordance between the standard commercial device and the smartwatch even in the different subgroups (lung disease, cardiovascular disease) for both parameters, SpO_2_ and HR (Figure S1).

## 4. Discussion

The major result of the present study is that the measurements of SpO_2_ obtained using the Apple Watch are reliable compared to the standard pulse oximetry technique in patients with cardiovascular disease, lung disease, and healthy subjects. It has been estimated that the number of Apple Watches in use worldwide is about 100 million [15]. These smartwatches are used today in cardiology, especially for the accurate and reliable diagnosis of atrial fibrillation [4,5,6]. Furthermore, the possibility of measuring changes in the ST segment has recently been demonstrated in our laboratory for the first time [7]. In this study, the watch was placed in different body positions to obtain nine bipolar ECG tracings (corresponding to Einthoven leads I, II, and III). The multichannel smartwatch ECG reliably identified ST-segment changes in patients with acute coronary syndromes (NSTEMI and STEMI). Finally, we and others demonstrated that the smartwatches could be used in measuring the QT interval [8,9] and even Brugada syndrome [10]. As an additional feature, Apple developed the smartwatch Apple Watch 6 (Apple Inc, Cupertino, CA, USA) with a new sensor that consists of four LED clusters and four photodiodes. Incorporated into a completely redesigned crystal, this new sensor works in concert with the blood oxygen app to determine blood oxygen levels. Green, red, and infrared LEDs shine light onto the blood vessels in the wrist, and photodiodes measure the amount of light reflected. A recent study by Pipek et al. in outpatients with chronic obstructive pulmonary disease or interstitial lung diseases observed positive correlations between the Apple Watch device and commercial oximeters when evaluating heart rate measurements (r = 0.995, *p* < 0.001) and oximetry measurements (r = 0.81, *p* < 0.001) [16]. Our study is larger and improves on prior studies on this topic by including patients with cardiovascular disease. However, in contrast with the study by Pipek et al. [16], our study did not demonstrate differences in mean values of SpO_2_ measured with the Apple Watch compared to standard oximeters (Figure 3a). Therefore, our data did not show higher values with the Apple Watch compared to standard oximeters. Our data demonstrated a very good concordance between the SpO_2_ measured by the smartwatch compared with the standard commercial device (bias, −0.2289; SD, 1.66; lower limit, −3.49; and upper limit, 3.04). Therefore, the continuous monitoring of blood oxygen saturation with the wearable device assessed in the present study can be beneficial in various settings, both in patients with cardiovascular or lung diseases and in healthy subjects.

There are several limitations of the present report. In our study, even under ideal conditions, in a small percentage of cases (eight subjects) it was not possible to measure oxygen levels with the smartwatch. Skin perfusion, the anatomical variability of the wrist, and other reasons could be responsible. The data from our study were acquired in a well-controlled environment with a constant room temperature (20 ± 2 °C). Therefore, these results might not apply to different temperatures and environments—for example, in cold or hot temperatures. Another significant limitation is the lack of laboratory data as well as the fact that there were few subjects with saturation < 90% or with heart rate > 100/min. The Apple Watch was used by an expert medical operator and the data accuracy might not apply to a broad population of users. Permanent or temporary changes to the skin, such as some tattoos, are another factor that can affect measurements. The ink used in some tattoos, as well as their design and saturation, can block light from the sensor, preventing the O_2_ levels app from taking measurements. The accuracy of pulse oximetry can be influenced by multiple factors, including perfusion and skin pigmentation [17]. In our population, however, all subjects were white and without tattoos in the skin area used for smartwatch use.

## 5. Conclusions

In conclusion, our study demonstrates that the wearable device used in the present study could be used to assess SpO_2_ in patients with cardiovascular or lung diseases and in healthy subjects.

## Figures and Tables

**Table 1:** Participant characteristics.

	Healthy Subjects (*n* = 56)	Lung Disease (*n* = 60)	CV Disease (*n* = 141)	*p*
Age, y ± SD	43.18 ± 14.31	71.23 ± 10.44	69.21 ± 11.53	<0.001
Male, *n* (%)	24 (42.9)	45 (75)	99 (70.2)	<0.001
Weight, *n* ± SD	69.52 ± 12.15	77.59 ± 17.35	76.22 ± 15.08	<0.02
Height, *n* ± SD	168.30 ± 9.05	166.47 ± 7.37	165.51 ± 7.93	0.25
BMI, *n* ± SD	24.49 ± 3.64	27.90 ± 5.41	27.71 ± 4.50	<0.02
Hypertension, *n* (%)	10 (17.9)	52 (86.7)	126 (89.4)	<0.001
Diabetes mellitus, *n* (%)	4 (7.1)	21 (35)	47 (33.3)	<0.001
Dyslipidemia, n (%)	7 (12.5)	29 (48.3)	123 (87.2)	<0.001
ACS, *n* (%)	0 (0)	1 (1.7)	50 (35.5)	<0.001
CCS, *n* (%)	0 (0)	9 (15)	64 (45.4)	<0.001
Stroke/TIA, *n* (%)	0 (0)	3 (5)	6 (4.3)	<0.001
Smoke, *n* (%)	15 (26.8)	6 (10.0)	22 (15.6)	<0.001
COPD, *n* (%)	0 (0)	35 (58.3)	16 (11.3)	<0.001
OSAS, *n* (%)	0 (0)	16 (26.7)	10 (7.1)	<0.001
O_2_ therapy, *n* (%)	0 (0)	24 (40.0)	18 (12.8)	<0.001
Room temperature, *n* ± SD	21.79 ± 1.32	21.28 ± 0.55	21.32 ± 0.91	0.94
Body temperature, *n* ± SD	36.18 ± 0.36	36.20 ± 0.38	36.14 ± 0.40	0.98
Wrist circumference, *n* ± SD	16.15 ± 1.38	16.94 ± 1.15	17.03 ± 1.39	0.91

TIA = Transient Ischemic Attack; ACS = Acute Coronary Syndrome; CCS = Chronic Coronary Syndrome; BMI = Body Mass Index; OSAS = Obstructive Sleep Apnea Syndrome; COPD = Chronic Obstructive Pulmonary Disease.

## Data Availability

The data underlying this article will be shared upon reasonable request to the corresponding author.

## Supplementary Materials

The following supporting information can be downloaded at: https://www.mdpi.com/article/10.3390/jcm11061467/s1, Figure S1. Agreements and concordance between the standard commercial device and the smartwatch in the different subgroups. Table S1. SpO_2_ Correlation between the smartwatch and the standard commercial device in different subgroups. Table S2. SpO_2_ Concordance between the smartwatch and the standard commercial device in difference subgroups. Table S3. HR Correlation between the smartwatch and the standard commercial device in different subgroups. Table S4. HR Concordance between the smartwatch and the standard commercial device in difference subgroups.
