# Supplementary material for: Assessment of Non-Invasive Measurements of Oxygen Saturation and Heart Rate with an Apple Smartwatch: Comparison with a Standard Pulse Oximeter
Source: J Clin Med. 2022 Mar 8;11(6):1467. doi: 10.3390/jcm11061467 (PMC8951323; doi:10.3390/jcm11061467)
Supplement: Supplementary file 1 [file jcm-11-01467-s001.zip › jcm-1599002-supplementary file S1.pdf]

Supplemental File Figure S1

SpO$_2$ Correlation in patients with lung disease

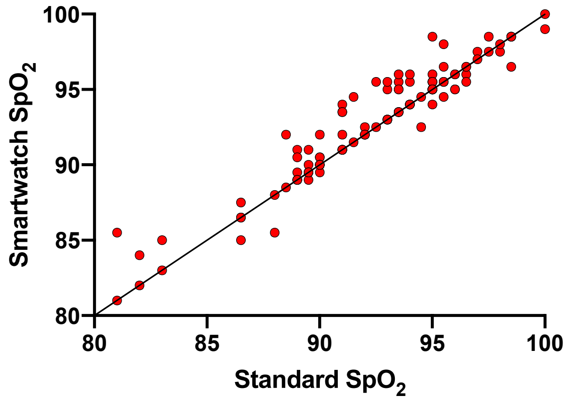

R=0.85

SpO$_2$ Correlation in patients with CV disease

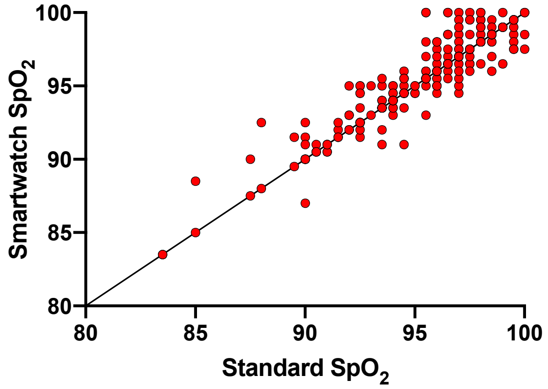

R=0.74

HR Correlation in patients with lung disease

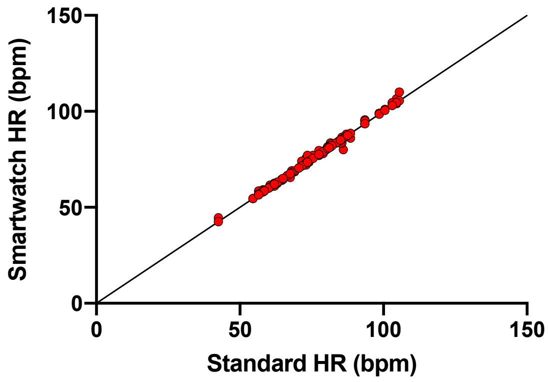

R=0.99

HR Correlation in patients with CV disease

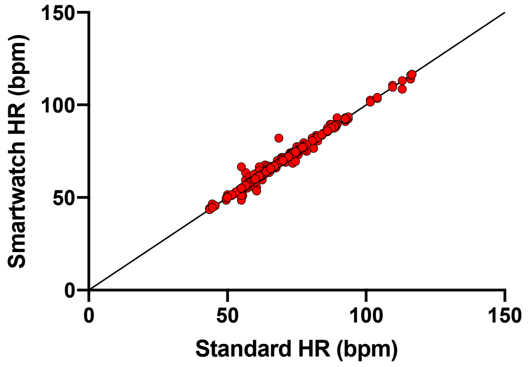

R=0,9833

Figure S1. Agreements and concordance between the standard commercial device and the smartwatch in the different subgroups.
