# Supplementary material for: Assessment of Non-Invasive Measurements of Oxygen Saturation and Heart Rate with an Apple Smartwatch: Comparison with a Standard Pulse Oximeter
Source: J Clin Med. 2022 Mar 8;11(6):1467. doi: 10.3390/jcm11061467 (PMC8951323; doi:10.3390/jcm11061467)
Supplement: Supplementary file 1 [file jcm-11-01467-s001.zip › jcm-1599002-supplementary file S2.pdf]

Supplemental file S2

Sup Table S1

SpO_2 Correlation between the smartwatch and the standard commercial device in different subgroups

	Healthy subjects	Lung Disease	CV Disease
**r**	0.91	0.93	0.86
**95% CI**	0.85-0.93	0.87-0.95	0.81-0.89
**P**	<0.0001	<0.0001	<0.0001

Sup Table S2

SpO_2 Concordance between the smartwatch and the standard commercial device in difference subgroups

	Healthy subjects	Lung Disease	CV Disease
**bias**	-0.84	-0.86	-0.41
**SD of bias**	1.51	1.55	1.58
**95% CI**	-3.6–2.3	-3.8-2.2	-3.5-2.7

Sup Table S3

HR Correlation between the smartwatch and the standard commercial device in different subgroups

	Healthy subjects	Lung Disease	CV Disease
**r**	0.98	0.99	0.98
**95% CI**	0.97-0.99	0.99-0.99	0.97-0.99
**P**	<0.0001	<0.0001	<0.0001

Sup Table S4

HR Concordance between the smartwatch and the standard commercial device in difference subgroups

	Healthy subjects	Lung Disease	CV Disease
**bias**	-0.15	-0.38	-0.11
**SD of bias**	2.3	1.53	2.5
**95% CI**	-4.9–4.7	-3.4-2.6	-5-4.8
